# Optimising computer aided detection to identify intra-thoracic tuberculosis on chest x-ray in South African children

**DOI:** 10.1371/journal.pgph.0001799

**Published:** 2023-05-16

**Authors:** Megan Palmer, James A. Seddon, Marieke M. van der Zalm, Anneke C. Hesseling, Pierre Goussard, H. Simon Schaaf, Julie Morrison, Bram van Ginneken, Jaime Melendez, Elisabetta Walters, Keelin Murphy

**Affiliations:** 1 Faculty of Medicine and Health Sciences, Department of Paediatrics and Child Health, Demond Tutu TB Centre, Stellenbosch University, Cape Town, South Africa; 2 Department of Infectious Disease, Imperial College London, London, United Kingdom; 3 Faculty of Medicine and Health Sciences, Department of Paediatrics and Child Health, Stellenbosch University, Stellenbosch, South Africa; 4 Radboud University Medical Center, Nijmegen, The Netherlands; 5 Delft Imaging, ‘s-Hertogenbosch, The Netherlands; 6 Newcastle-upon-Tyne NHS Foundation Trust, Newcastle upon Tyne, United Kingdom; University of California Irvine, UNITED STATES

## Abstract

Diagnostic tools for paediatric tuberculosis remain limited, with heavy reliance on clinical algorithms which include chest x-ray. Computer aided detection (CAD) for tuberculosis on chest x-ray has shown promise in adults. We aimed to measure and optimise the performance of an adult CAD system, CAD4TB, to identify tuberculosis on chest x-rays from children with presumptive tuberculosis. Chest x-rays from 620 children <13 years enrolled in a prospective observational diagnostic study in South Africa, were evaluated. All chest x-rays were read by a panel of expert readers who attributed each with a radiological reference of either ‘tuberculosis’ or ‘not tuberculosis’. Of the 525 chest x-rays included in this analysis, 80 (40 with a reference of ‘tuberculosis’ and 40 with ‘not tuberculosis’) were allocated to an independent test set. The remainder made up the training set. The performance of CAD4TB to identify ‘tuberculosis’ versus ‘not tuberculosis’ on chest x-ray against the radiological reference read was calculated. The CAD4TB software was then fine-tuned using the paediatric training set. We compared the performance of the fine-tuned model to the original model. Our findings were that the area under the receiver operating characteristic curve (AUC) of the original CAD4TB model, prior to fine-tuning, was 0.58. After fine-tuning there was an improvement in the AUC to 0.72 (p = 0.0016). In this first-ever description of the use of CAD to identify tuberculosis on chest x-ray in children, we demonstrate a significant improvement in the performance of CAD4TB after fine-tuning with a set of well-characterised paediatric chest x-rays. CAD has the potential to be a useful additional diagnostic tool for paediatric tuberculosis. We recommend replicating the methods we describe using a larger chest x-ray dataset from a more diverse population and evaluating the potential role of CAD to replace a human-read chest x-ray within treatment-decision algorithms for paediatric tuberculosis.

## Background

An estimated 230,000 children (<15 years) died from tuberculosis in 2020 [1]. This mortality is largely attributable to delays in access to diagnosis and treatment and can only be reduced through the development of new diagnostic tools and strategies. Currently, chest X-ray remains widely used within clinical treatment-decision algorithms for paediatric tuberculosis, particularly in cases where microbiological tests are either negative or unavailable [2–4]. Chest x-rays are the most accessible imaging modality in low resource countries. Their value, however, is limited by variability in inter-reader agreement and lack of access to specialist interpretation [5–7].

There is increasing evidence from adult populations that computer aided detection (CAD) for tuberculosis on chest x-ray can be used to screen for tuberculosis to improve case detection [8–10]. In 2021, the World Health Organization (WHO) approved the use of CAD for tuberculosis screening and triage programs in people older than 15 years [11]. While some CAD software products are marketed for use in children from the age of 4 years, there are currently no publicly available data on their performance in this age group. Given the limited availability of accurate diagnostic tools for tuberculosis in children, developing CAD solutions for this population should be prioritized.

While the increasing evidence around the use of CAD in adults is encouraging, there are some unique challenges around CAD development in paediatrics. The spectrum of radiological disease in children is wider than in adults and chest x-ray features are more diverse [12,13]. While adult-type tuberculosis predominantly involves the lung parenchyma, the hallmark radiological feature of paediatric tuberculosis is enlargement of intra-thoracic lymph nodes, a feature that is rarely seen in adults [14–16]. Intra-thoracic lymph node disease can occur in the absence of lung parenchymal disease and may only be evident through the presence of narrowing or deviation of the large airways. While current CAD algorithms have demonstrated performance in identifying lung parenchymal abnormalities, their ability to identify abnormalities involving the intra-thoracic lymph nodes and large airways is not known. Additional considerations in a paediatric population include CAD systems having been trained using adult posteroanterior (PA) views whereas anteroposterior (AP) views are more common in young children, difficulties around the acquisition of good quality chest x-rays in this age group and differences in the normal anatomy of the chest across the paediatric age spectrum.

Acknowledging these challenges, we hypothesise that current CAD systems may underperform in children and will require further optimisation with paediatric chest x-ray data. We therefore set out to evaluate the diagnostic performance of CAD4TB, a well-known commercial artificial intelligence (AI) system, for the identification of tuberculosis on chest x-rays taken from a cohort of young, symptomatic children investigated for intra-thoracic tuberculosis in South Africa. We then sought to adapt the software on paediatric chest x-rays to optimise performance with the aim of exploring whether a CAD chest x-ray read has the diagnostic potential to replace a human chest x-ray read within current diagnostic treatment-decision algorithms for paediatric tuberculosis.

## Methods

### Ethics statement

The chest x-rays used in this study were taken from children enrolled into a prospective diagnostic study in Cape Town, South Africa between April 2012 and March 2017 [17]. The study was approved by the Stellenbosch University Health Research Ethics Committee (N11/09/282), local hospitals and the provincial Department of Health and all children were enrolled with the written informed consent of their parents or legal caregivers.

### Cohort description

Children <13 years were enrolled on the diagnostic study as presumptive tuberculosis cases, using standard eligibility criteria with either well-defined symptoms of tuberculosis (cough >2 weeks, fever >1 week, weight loss) or with acute symptoms (<2 weeks) and other additional risk factors for tuberculosis disease (exposure to an adult tuberculosis source case, a positive tuberculin skin test or a chest x-ray considered by the enrolling doctor as being suggestive of tuberculosis). All children were systematically investigated for tuberculosis at the time of enrolment, which included the collection of multiple respiratory samples and a frontal (AP or PA, depending on the child’s age) chest x-ray with or without a lateral film. All chest x-rays were digital. Children were carefully followed up for 6 months or until treatment completion and were retrospectively classified using standard clinical case definitions as having ‘confirmed’, ‘unconfirmed’ or ‘unlikely’ tuberculosis [2].

### Methodology of chest x-ray classification by expert readers

Baseline chest x-rays were read in real-time by enrolling doctors to inform the decision to enrol on the study or not. Chest x-rays were also read retrospectively by at least 2 of a group of 4 specialist paediatricians with experience in chest x-ray interpretation for paediatric tuberculosis (expert readers) and classified using a consensus process. The expert readers were blinded to all clinical and microbiological information and to each other’s reads. Each chest x-ray was independently classified by at least 2 readers for technical acceptability and (if technically acceptable), as normal or not. Chest x-rays classified as abnormal were given a final overall classification of ‘tuberculosis’, ‘uncertain tuberculosis’ or ‘not tuberculosis’. If the following chest x-ray features were identified by the human reader then the chest x-ray was classified as ‘tuberculosis’: intra-thoracic lymphadenopathy (perihilar, paratracheal or subcarinal), compression or deviation of the large airways (bronchi or trachea), cavitary disease, a Ghon focus, miliary infiltrates, pleural effusion and expansile pneumonia. Chest x-rays with abnormal radiological features other than these, as well as those interpreted as normal, were classified as ‘not tuberculosis’. If the reader did not feel confident to categorise as ‘tuberculosis’ or ‘not tuberculosis’, then they classified the image as ‘uncertain tuberculosis’. All information was captured on standard chest x-ray case report forms. After independent interpretation by 2 readers, a final consensus classification for each chest x-ray was established for technical acceptability (acceptable or not) and radiological diagnostic certainty (‘tuberculosis’ or ‘not tuberculosis’). Where there was not consensus (on acceptability or diagnostic certainty) or when one of the first 2 readers classified the chest x-ray as ‘uncertain tuberculosis’, a third reader independently reviewed the chest x-ray; the majority read was taken as final. Inter-reader agreement on ‘tuberculosis’ versus ‘not tuberculosis’ was calculated using Cohen’s kappa co-efficient.

### CAD4TB methodology

CAD4TB is a commercially available AI system for detection of tuberculosis on chest x-ray and is licensed for use in adults and children above 4 years of age. In this work we use the most recently released version, v7. The system takes a frontal chest x-ray as input and produces a score in the range 0–100 indicating the likelihood of tuberculosis. It additionally produces a heatmap image to indicate which regions of the lungs are considered abnormal.

### Test and training set selection

From the available chest x-ray data, a test set of 80 images with consensus readings was selected for evaluation of the CAD4TB software before and after fine-tuning. The rest of the images (where consensus was achieved) were set aside as the training set. The test set images were selected according to the following criteria: 1) all 80 images had to have been read by at least the same unique Reader 1 and Reader 2 to enable evaluation of the software against unique individual experts, 2) all 80 images had to have been classified as technically acceptable by all readers, 3) exactly half of the images (40 images) had to have a radiological reference of ‘tuberculosis’ and 4) the proportion of chest x-rays from each of the specified age-groups in the test set had to be the same as in the training set. The age-groups selected, in months, were 0–5, 6–11, 12–23, 24–35, 36–47, and 48 months or older. A programmatic script was created to select images at random and add them to the test set (if doing so did not violate these conditions) until 80 images had been added.

### CAD4TB v7

The CAD4TB v7 system was run on the test set of 80 images, providing each one with a score in the range 0–100. These scores were used for receiver operating characteristic (ROC) analysis to evaluate the system performance at identifying paediatric tuberculosis on chest x-ray against the primary reference standard of chest x-ray set by the expert readers.

### CAD4TB v7 fine-tuning

In machine-learning terms, fine-tuning refers to the re-training of a system already trained with different data. The CAD4TB v7 system was fine-tuned with images not included in the test set, using the consensus human read as the training label for each image. During the training process of an AI system a percentage of the training data is used for validation (determining how well the system is performing as training continues), while the remainder is used for the training process itself. The performance on the validation set identifies the optimal point at which to stop training and prevent overfitting to the training data.

Based on the limited number of images available for fine-tuning it was decided to perform cross-validation training on the CAD4TB system, as follows: the training data was split into five different training data folds with 20% of each fold being reserved for validation. Each image was used as part of the validation set in exactly one of these five folds. The CAD4TB system was fine-tuned five times separately, using these data folds, with the optimal system selected each time based on the validation set performance. The mean score from these five systems is considered as the final score of the fine-tuned system. The scores are used for ROC analysis to evaluate the performance of the fine-tuned system.

### Evaluation

ROC analysis was performed using the python scikit-learn package and area under the ROC curve (AUC) was calculated. This was done for the CAD4TB scores and the scores of the fine-tuned system using several different reference standards as follows: 1) radiological reference of consensus human expert read, 2) clinical case definitions of ‘all tuberculosis’ (confirmed and unconfirmed) and ‘confirmed tuberculosis’ only, and 3) individual opinions of unique Readers 1 and 2. Statistical significance of different AUC values was calculated using DeLong’s test [18]. The CAD4TB scores before and after fine-tuning are plotted and sample heatmaps are illustrated.

## Findings

### Image classification

**Fig 1:**
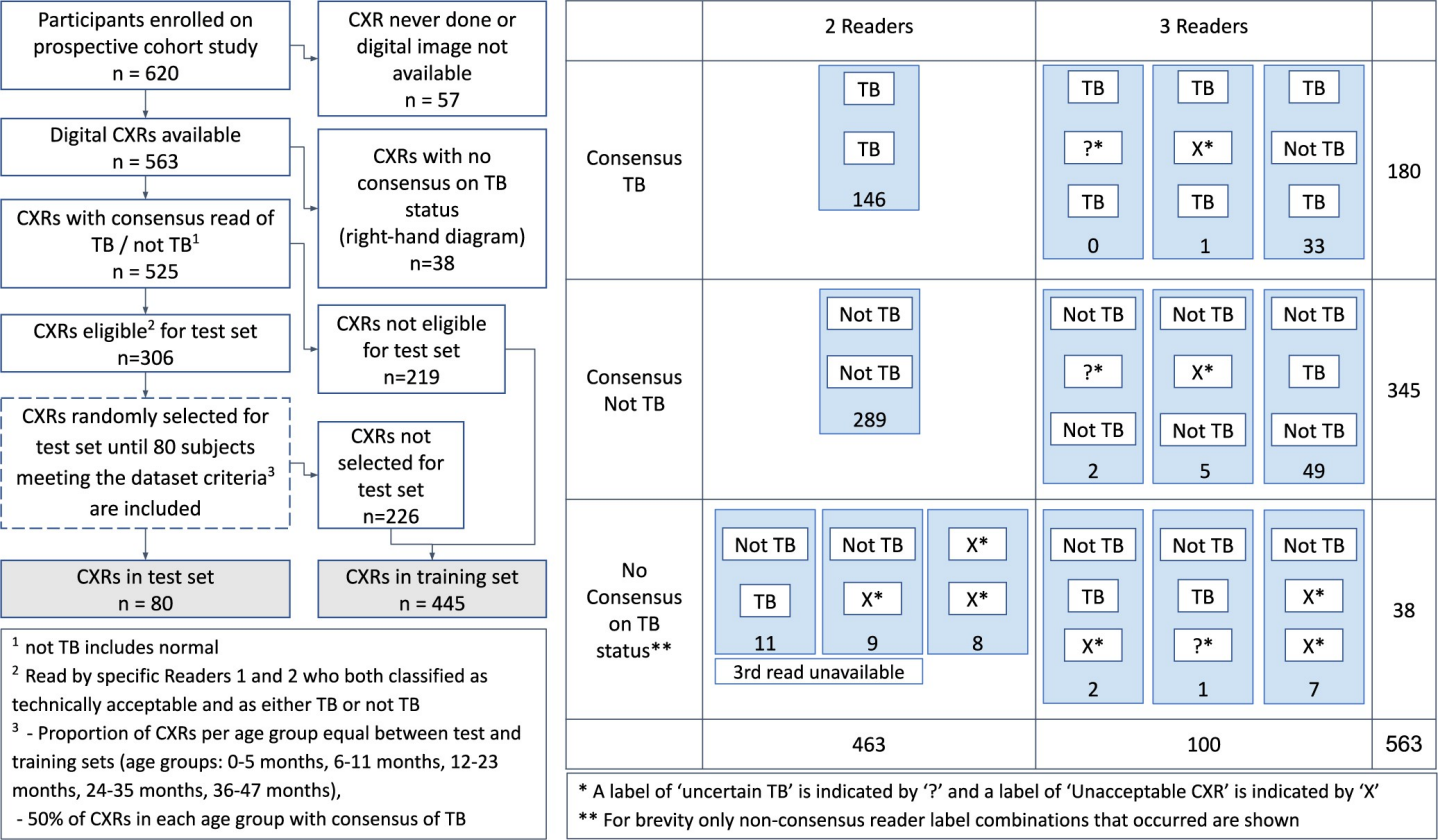
Data selection and consensus processes. Left: A flowchart illustrating how chest x-ray images were selected and assigned to the test or training sets. Right: An illustration of the possible reader label combinations that could lead to consensus of ‘tuberculosis’ (top row), ‘not tuberculosis’ (middle row) or ‘no consensus’ (bottom row). Numbers indicate the number of cases with this reader label combination. CXR: Chest x-ray; TB: Tuberculosis.

**Fig 2:**
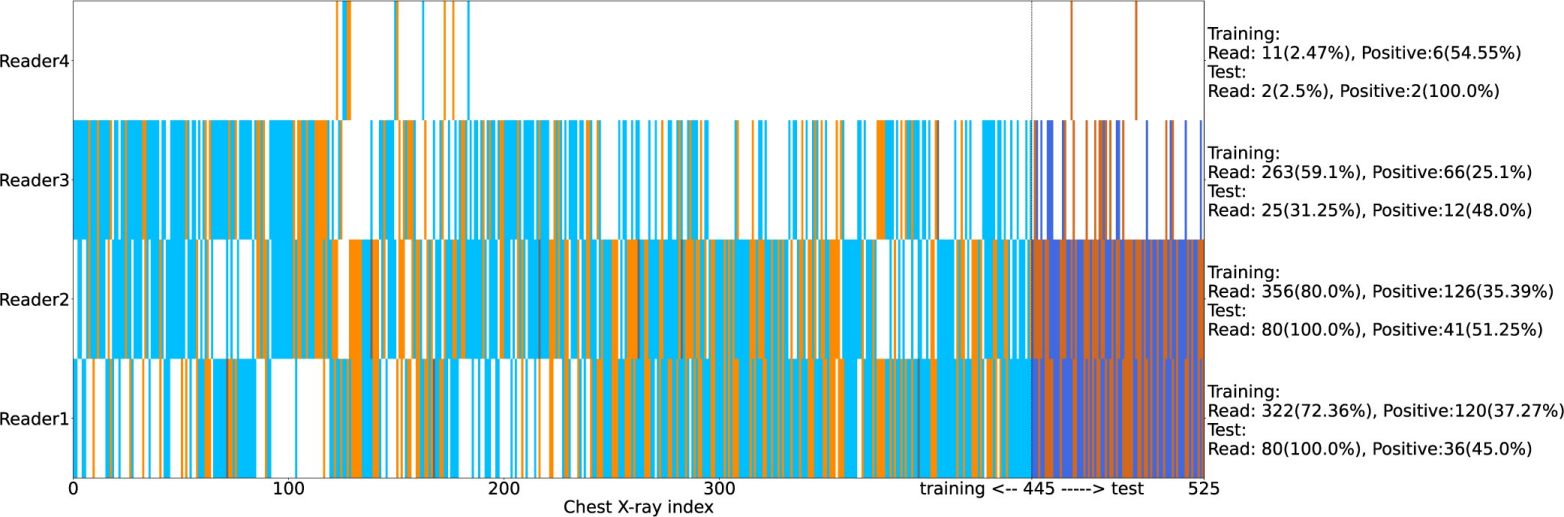
A visualization of number of chest x-ray reads per human reader and their assessment per image. The images are distributed on the X-axis with the test set to the right-hand side. A white region indicates the reader did not assess the image, blue indicates ‘not tuberculosis’, orange indicates ‘tuberculosis’, grey indicates either Uncceptable or Uncertain. Darker shades are used for the test set. The numbers and percentages of images read and images marked positive for ‘tuberculosis’ are provided per-reader on the right-hand side of the plot.

During recruitment to the diagnostic study, 620 children were enrolled. From these, 563 (90.8%) chest x-rays were available; 550/563 (98%) of the frontal images were AP. For 38/563 chest x-rays (6.7%) no consensus was reached on ‘tuberculosis’ versus ‘not tuberculosis’ after review by 2 or 3 readers. The final number of chest x-rays included in this analysis was 525. Fig 1 illustrates the process of data curation and training/test set selection (left) as well as the process of obtaining consensus readings (right). Fig 2 is a visual representation of the number of chest x-ray reads per reader and the number of chest x-rays classified as ‘tuberculosis’ or ‘not tuberculosis’ per reader, in the training and test sets. Reader 1 and Reader 2, who both read 100% of the chest x-rays in the test set, also each read a similar proportion of the training set– 322 and 356 chest x-rays respectively. Inter-reader agreement for ‘tuberculosis’ versus ‘not tuberculosis’ was: Reader 1 / Reader 2 (n = 307 where both readers read and gave score of ‘tuberculosis’ or ‘not tuberculosis’): kappa 0.48; Reader 1 / Reader 3 (n = 163 where both readers read and gave score of ‘tuberculosis’ or ‘not tuberculosis’): kappa 0.40; Reader 2 / Reader 3 (n = 199 where both readers read and gave score of ‘tuberculosis’ or ‘not tuberculosis’): kappa 0.58. Reader 4 was excluded due to the small number of chest x-rays read.

The cohort of children was young, with a median age of 17 months (interquartile range [IQR] 10–35 months) and 331 (63.0%) under the age of 2 years (Table 1). There were 66 (12.6%) children living with HIV and mean weight-for-age z-score was -1.68 [IQR -2.78–0.75]. Classified retrospectively, using standard clinical case definitions 263 (50.1%) children were tuberculosis cases (115 confirmed and 148 unconfirmed) and 241 (45.9%) were classified as having ‘unlikely tuberculosis’. For the remaining 21 (4.0%) no clinical classification was attributed due to missing follow-up data.

**Table 1:** Baseline demographics, clinical characteristics and final chest x-ray human consensus read, by test versus training set.

	All N = 525 n (%)	Training set N = 445 n (%)	Test set N = 80 n (%)
Age (months) Overall (median[interquartile range])	17[10–35]	17[10–35]	18[8.75–35.25]
0–5 months	75(14.3)	64(14.4)	11(13.8)
6–11 months	103(19.6)	87(19.6)	16(20.0)
12–23 months	153(29.1)	130(29.2)	23(28.8)
24–35 months	67(12.8)	57(12.8)	10(12.5)
36–48 months	38(7.2)	32(7.2)	6(7.5)
48+ months	89(17.0)	75(16.9)	14(17.5)
Sex			
Female	245(46.7)	211(47.4)	34(42.5)
Male	272(51.8)	230(51.7)	42(52.5)
Unknown	8(1.5)	4(0.9)	4(5.0)
HIV			
Positive	66 (12.6)	54 (12.1)	12 (15.0)
Negative	451 (85.9)	387 (86.9)	64 (80.0)
Unknown	8 (1.5)	4 (1.0)	4 (5.0)
WAZ (mean[interquartile range])	-1.68[-2.78–0.75]	-1.68[-2.78–0.72]	-1.67[-2.73–0.88]
Unknown	8 (1.5)	4(0.9)	4(5.0)
Tuberculosis case classification			
Confirmed tuberculosis	115 (21.9)	93 (20.9)	22 (27.5)
Unconfirmed tuberculosis	148 (28.2)	127 (28.5)	21 (26.3)
Unlikely tuberculosis	241 (45.9)	210 (47.2)	31 (38.7)
Not Available^a^	21 (4.0)	15 (3.4)	6 (7.5)
Human consensus read chest x-ray classification			
Not tuberculosis^b^	345 (65.7)	305 (68.5)	40 (50.0)
Tuberculosis	180 (34.3)	140 (31.5)	40 (50.0)

WAZ–weight for age z-score, HIV–Human Immunodeficiency Virus.

^a^Cases that could not be classified using standard clinical case definitions.

^b^Includes normal chest x-rays.

### Performance of CAD4TB to identify tuberculosis on chest x-ray before and after fine-tuning using the reference of the consensus human expert read

**Fig 3:**
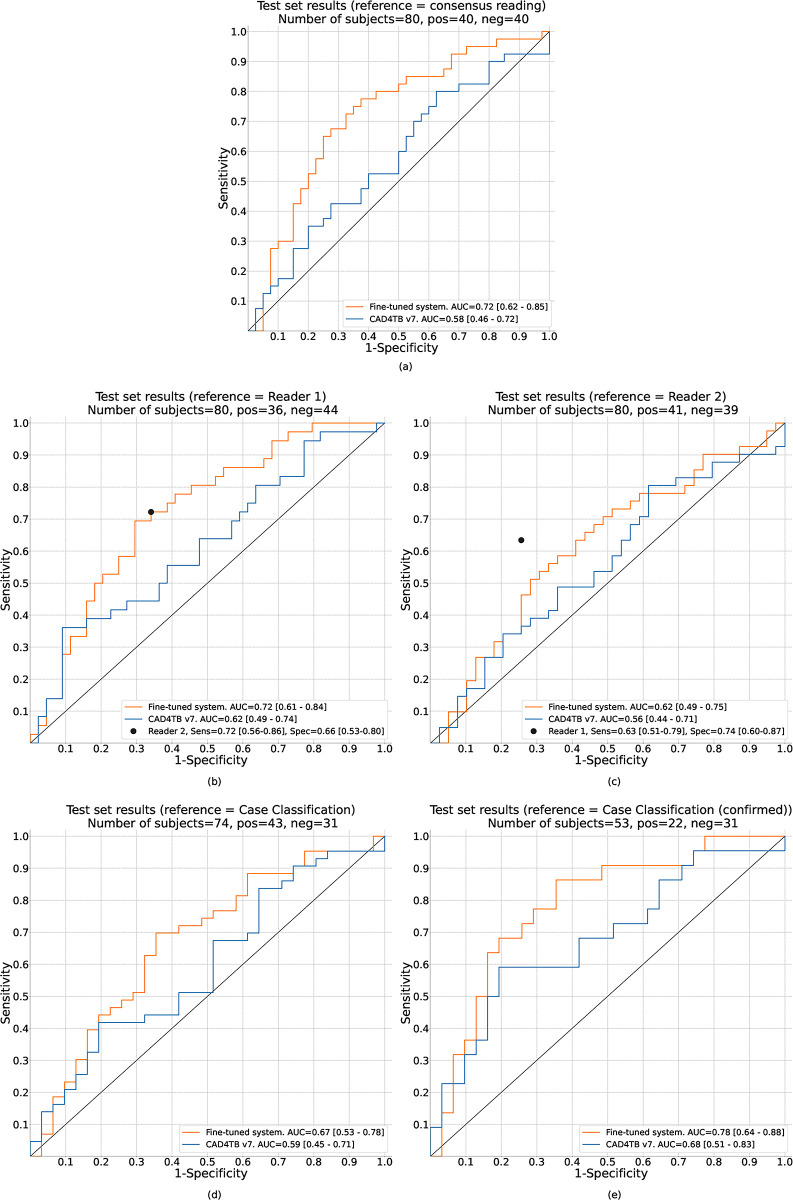
ROC curves illustrating the performance of CAD4TB v7.0 before and after fine-tuning against different reference standards. The numbers in square brackets indicate the 95% confidence intervals which were computed by bootstrapping. Fig 3A: Performance of CAD4TB v7 before and after fine-tuning with paediatric data. The reference standard is the consensus radiological reading of 2 or more expert readers. Fig 3B and 3C: ROC curves using individual readers as the reference standard and illustrating the performance of the second reader in each case. The black dots show the sensitivity and specificity of the individual Reader 2 (Fig 3B) and individual Reader 1 (Fig 3C), compared to the reference of individual reader 1 and individual reader 2 respectively, in each case. Fig 3D and 3e: Performance of CAD4TB using final clinical case classification (confirmed tuberculosis, unconfirmed tuberculosis, unlikely tuberculosis). ‘All tuberculosis’ (confirmed and unconfirmed) is the reference standard in Fig 3D (this information was not available for 6 test set subjects) and ‘confirmed tuberculosis’ (with ‘unconfirmed’ tuberculosis cases excluded) is the reference standard in Fig 3E.

Using the CAD4TB v7 system, prior to fine-tuning, and compared to the consensus human expert reading, the software had an AUC of 0.58 (95%CI: 0.46–0.72). After fine-tuning the CAD4TB v7 system with the 445 chest x-rays in the training set there was a significant improvement in the AUC to 0.72 (95%CI: 0.62–0.85; p = 0.0016; Fig 3A).

### Performance of CAD4TB and each human expert reader

Using unique Reader 1 as the reference standard (Fig 3B), fine-tuning improved the AUC from 0.62 (95%CI: 0.49–0.74) to 0.72 (95%CI: 0.61–0.84; p = 0.03). The fine-tuned system had a performance equivalent to unique Reader 2 (sensitivity = 0.72, specificity = 0.66) against this reference standard. Using Reader 2 as the reference standard the AUC of the fine-tuned system (0.62; 95%CI: 0.49–0.75) was not statistically different to that of the original CAD4TB system (0.56; 95%CI: 0.44–0.71; p = 0.27) (Fig 3C). The fine-tuned system did not reach the sensitivity (0.63) and specificity (0.74) of Reader 1 in this case.

### Performance of CAD4TB to identify children classified as having tuberculosis using standard clinical case definitions

Compared to the clinical case definitions of ‘all tuberculosis’ (confirmed and unconfirmed) the AUC increased from 0.59 (95%CI: 0.45–0.71) to 0.67 (95%CI: 0.53–0.78; p = 0.12) after fine-tuning. When compared to ‘confirmed tuberculosis’ only, it increased from 0.68 (95%CI: 0.51–0.83) to 0.78 (95%CI: 0.64–0.88; p = 0.10) (Fig 3D and 3E).

### Distribution of CAD4TB scores before and after fine-tuning

**Fig 4:**
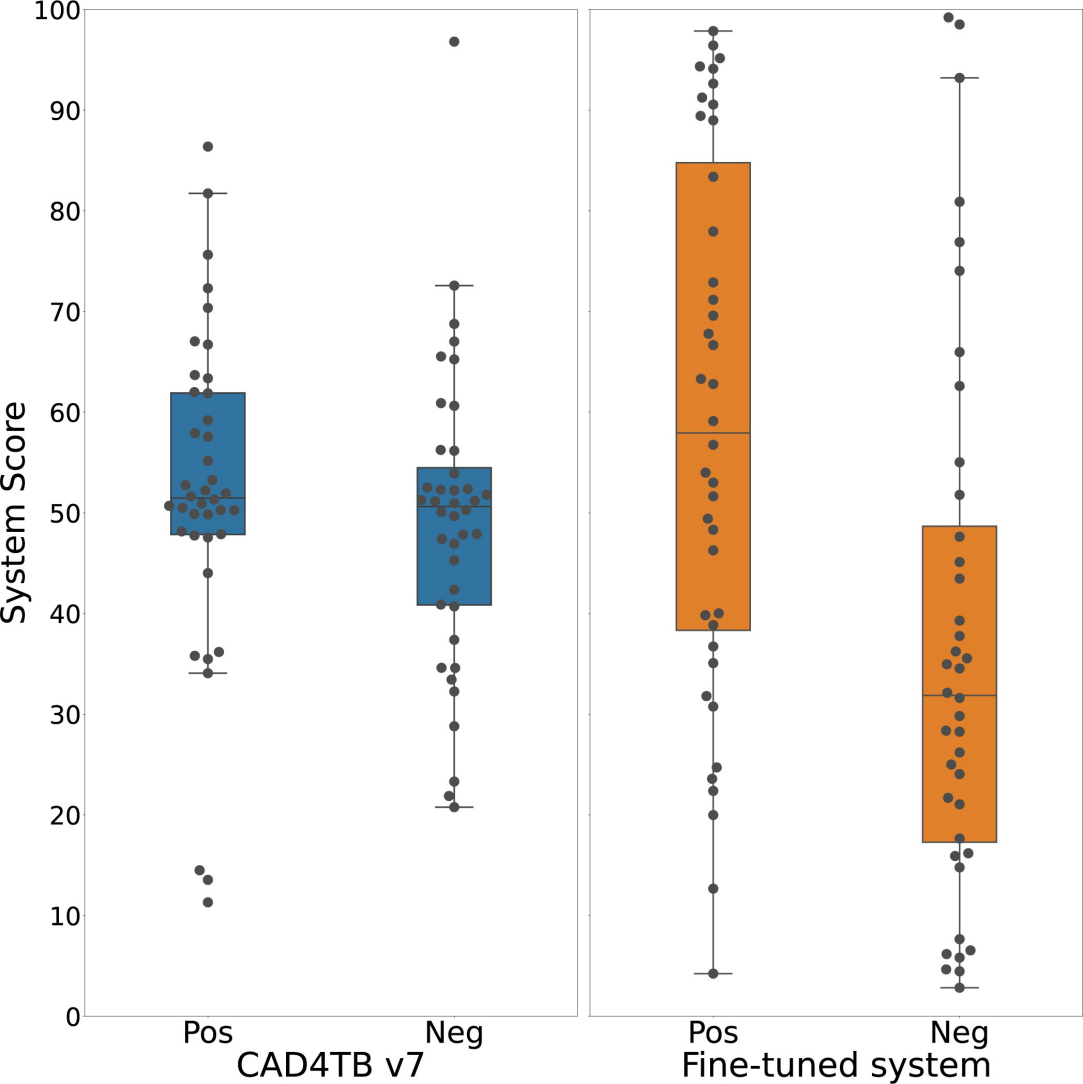
Boxplots illustrating the distribution of test-set scores (n = 80) for chest x-rays classified as ‘tuberculosis’ as per radiological reference read (“Pos”) and chest x-rays classified as ‘not tuberculosis’ (“Neg”). Left: Scores from the CAD4TB v7 system before re-training. Right: Scores from the system re-trained with paediatric data.

Prior to fine-tuning, the ranges of CAD4TB scores for chest x-rays classified as ‘tuberculosis’ and ‘not tuberculosis’ by the human expert panel were similar; median 51.5 (IQR 47.8–61.9) and median 50.6 (IQR: 40.8–54.5) respectively. After fine-tuning, the median score for chest x-rays classified as ‘tuberculosis’ was 57.9 (IQR 38.3–84.8) and for chest x-rays classified as ‘not tuberculosis’ was 31.8 (IQR 17.3–48.7) (Fig 4).

### Heatmaps

**Fig 5:**
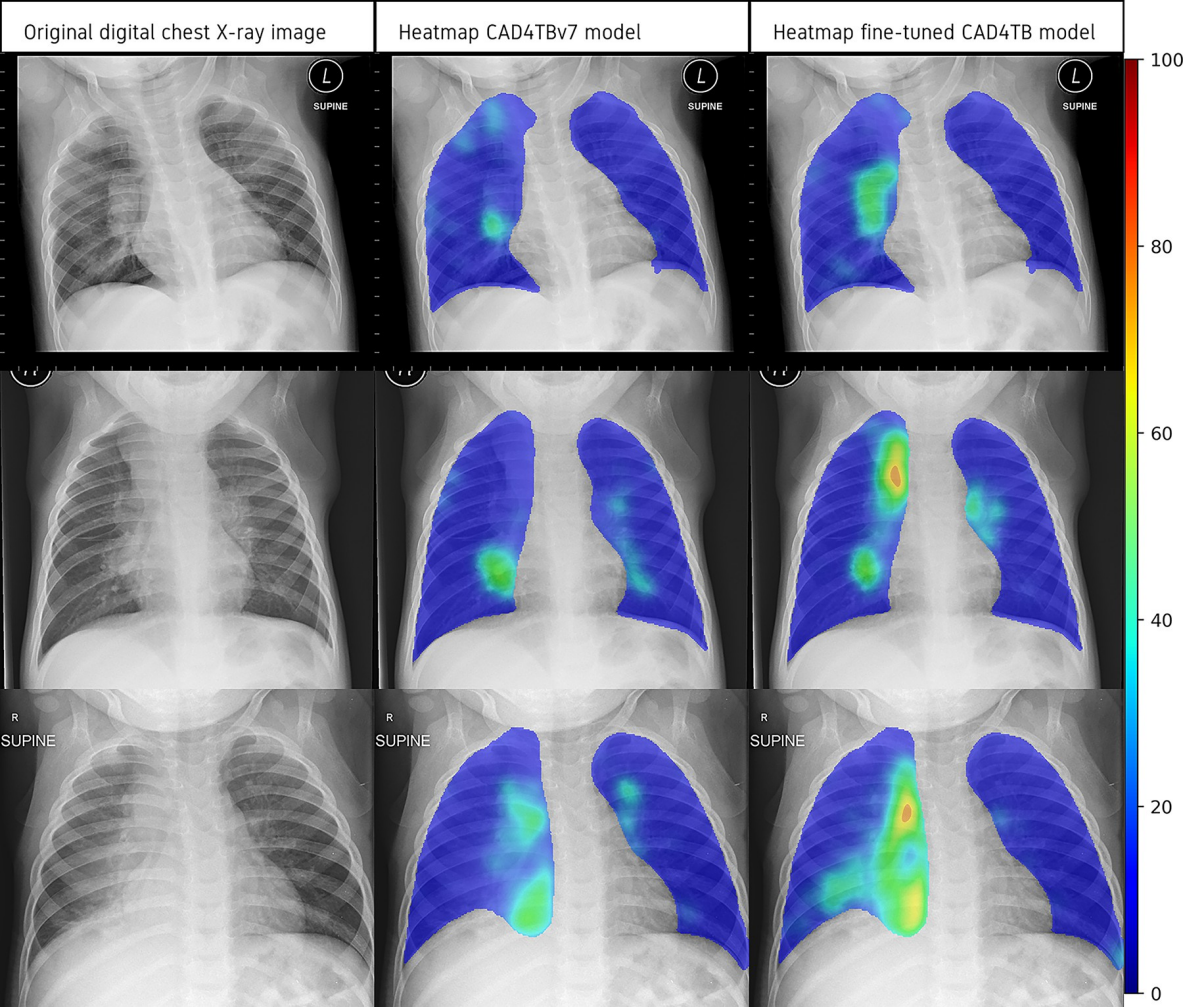
Digital chest x-ray images and the associated heatmaps generated by the CAD4TB v7 model and the fine-tuned CAD4TB model. The colour-bar to the right indicates the level of abnormality where 0 is normal and 100 is most abnormal.

Fig 5 is a set of 3 digital chest x-ray images and the associated heatmaps generated by the CAD4TB v7 model and the fine-tuned CAD4TB model. These images demonstrate the improvements seen in the fine-tuned CAD4TB model over the CAD4TB v7 model in its capability to identify enhancement in the perihilar and paratracheal regions of the lungs; chest x-ray features which suggest the presence of intra-thoracic lymphadenopathy.

## Interpretation

This is the first description of the performance of CAD for the detection of ‘tuberculosis’ on chest x-rays in children. We have shown that, through fine-tuning CAD4TB v7 with a set of well characterised and labelled paediatric chest x-rays, performance could be significantly improved. Our fine-tuned CAD4TB model achieved an AUC of 0.72 against the radiological reference standard of a consensus human expert read. Against the reference standard of clinically diagnosed and microbiologically confirmed tuberculosis (with unconfirmed tuberculosis excluded) our fine-tuned model achieved an AUC of 0.67 and 0.78 respectively. The distribution of CAD scores between chest x-rays classified as ‘tuberculosis’ and ‘not tuberculosis’ by the human expert panel also improved after fine-tuning, suggesting that the fine-tuning process improved the CAD system’s discriminative power.

Given the limited data on CAD for paediatric tuberculosis, and the absence of data on CAD’s diagnostic performance in this context, we compare our findings to those from paediatric pneumonia studies [19–22]. Two analyses of CAD performance for pneumonia using chest x-rays from the PERCH study reported AUCs of 0.93 and 0.85 against the radiological reference standard of a consensus human read [21,22]. The use-case of CAD for pneumonia is further advanced than for paediatric tuberculosis and there is wider access to larger chest x-ray datasets. It is thus not surprising that CAD’s performance for pneumonia exceeds what we report. Given that the radiological features of pneumonia are well-defined and not as diverse as paediatric tuberculosis the CAD learning process for pneumonia is likely to be less complex. The radiological features of pneumonia are largely seen in the lung parenchyma, the chest x-ray area which has been the focus of most CAD learning, while in paediatric tuberculosis important radiological abnormalities, such as intra-thoracic lymphadenopathy, lie outside this area and within the mediastinum.

Chen and colleagues demonstrated that CAD for pneumonia performed better on a chest x-ray dataset that was classified with high concordance between human readers–there was a 14% reduction in CAD performance for low concordance images [21]. In our study inter-reader agreement on ‘tuberculosis’ versus ‘not tuberculosis’ was fair to moderate between our readers. Although this is comparatively good in the context of chest x-ray interpretation for paediatric tuberculosis, the inter-reader agreement for pneumonia is generally better [7,14,21,23,24]. The poorer concordance for features of tuberculosis compared to pneumonia on chest x-ray indicates that the task of identifying tuberculosis is more difficult, which could inherently limit CAD’s performance in this context.

The radiological reference standard of a consensus human read chest x-ray was chosen for this analysis after careful consideration of the proposed role of CAD in the treatment decision pathway for paediatric tuberculosis. There is currently no single diagnostic test with acceptable accuracy for diagnosing tuberculosis in children. For now, and the foreseeable future, the reliance on clinical treatment-decision algorithms in this population is likely to remain. Additionally, the radiological spectrum of tuberculosis disease in children is wide and varies with age. Even the most optimised chest x-ray evaluation system will likely always remain as one component of the paediatric treatment-decision algorithm. Acknowledging this context, we aimed to evaluate whether CAD could replace a human read chest x-ray rather than to evaluate whether CAD could be a single gold-standard test for paediatric tuberculosis. If CAD’s performance could be optimised to be as good as a human expert reader, then this could provide access to automated chest x-ray interpretation with equivalent accuracy to a specialist doctor even in remote areas.

We acknowledge the limitations around using human read chest x-ray as a reference standard. One of these is suboptimal inter-reader agreement. To evaluate this, and cognisant that in clinical practice chest x-rays are usually read by one reader only, we also carried out an analysis using individual chest x-ray readers as the reference. This demonstrates how the CAD system would perform as a replacement for a single reader in such scenarios. Fig 3B and 3C illustrates that the CAD system performs as well as an independent human expert (Reader 2) when Reader 1 is used as the reference standard, but has a weaker performance when Reader 2 is used as the reference standard. We found no systematic reason for this finding, and hypothesize that this can be partially explained by inter-reader disagreement, which occurred in 25 of the 80 images in the test set and is a widely acknowledged limitation of chest x-ray interpretation by human readers [7,14,23,24]. Alternate reference standards were considered. A gold-standard imaging reference standard such as computed tomography (CT) of the chest, although optimal, was not available. While chest x-rays labelled with the microbiological reference of ‘confirmed tuberculosis’ (*Mycobacterium tuberculosis* positive either on culture or Xpert MTB/RIF) would have provided the highest level of certainty of tuberculosis disease, this approach would have excluded 56% (148/263) of the tuberculosis cases (those with unconfirmed tuberculosis) from the training process. Paediatric tuberculosis represents a disease spectrum ranging from early, non-severe disease (where microbiological confirmation rates are low) to more advanced and severe disease [25–27]. Similarly, the radiological features vary across this spectrum and an optimal CAD solution should be trained to identify chest x-rays from children with both microbiologically confirmed and unconfirmed disease.

Because the chest x-ray dataset used in this analysis was collected from a diagnostic study and not a tuberculosis screening study, the definition of ‘tuberculosis’ on chest x-ray was weighted towards specificity and excluded consolidation as an isolated radiological feature. Enrolled children were symptomatic, and the aim of the chest x-ray interpretation was to identify features that distinguished pulmonary tuberculosis from other respiratory pathology, such as viral and bacterial respiratory tract infections. This contrasts with the role of chest x-ray in tuberculosis screening studies where sensitivity is prioritised and chest x-rays are primarily assessed for any abnormality versus normal. Only 11% of the chest x-rays in this dataset were classified as normal by the expert panel. We were able to show a meaningful improvement in CAD performance after fine-tuning to distinguish ‘tuberculosis’ from ‘not tuberculosis’ in a chest x-ray dataset where the ‘not tuberculosis’ chest x-rays were predominantly abnormal which is promising. We hypothesize that CAD will perform better in a tuberculosis screening context where most chest x-rays are normal and the radiological difference between ‘tuberculosis’ and ‘not tuberculosis’ is wider.

Confidence in the reported performance of various CAD models is compromised when there is a lack of transparency around the methodology used to train the software. Our analysis is strengthened by the fact that the chest x-rays were collected from a rigorous diagnostic study so that the certainty of tuberculosis disease amongst the cases and the absence of tuberculosis disease amongst those classified as ‘unlikely tuberculosis’ was high. We used original digital images, minimising the potential reduction in image quality through the digitization of analogue chest x-rays. The expert readers were specialist paediatricians with specific experience in interpreting chest x-rays from children with presumptive tuberculosis in a tuberculosis-endemic setting.

Our work has limitations. The number of children in the diagnostic study was sufficient for performing diagnostic analyses, but the chest x-ray dataset was small in the context of deep learning. Data were collected from one site and from children who were investigated for tuberculosis at hospital-level. Consequently, their disease spectrum and severity profile may not reflect most children with tuberculosis, who are typically diagnosed in primary health care with less severe disease. The human readers had access to both frontal and lateral views while the CAD4TB software only analyses frontal chest x-rays. This may have contributed to some of the discrepancies between the human readers and CAD4TB, as enlarged intra-thoracic lymph nodes may be more clearly seen on the lateral view. Although we endeavoured to include the chest x-rays from as many of the children enrolled in the diagnostic study as possible, 38 of the available chest x-rays were excluded since consensus on the radiological reference standard by the expert panel could not be achieved. In 15 of these cases at least 2 readers reported the chest x-ray as being technically unacceptable, which we consider a necessary reason for exclusion. For the remaining 23 chest x-rays a third reader was required and that read was either unavailable (n = 20) or inconclusive (n = 3). These 23 cases are likely to be challenging, since they did require a third read, and therefore their exclusion could be considered as a spectrum bias, reducing the complexity of our final dataset. However, we successfully included 90 cases where 3 reads were required for consensus and we believe that the final dataset has a representative and realistic balance of straightforward and more complex cases.

Using confirmed tuberculosis as the reference standard (and with unconfirmed tuberculosis excluded), our fine-tuned model reached a specificity of 79% at a sensitivity of 66%. Although this falls below the WHO’s target product profile for both a ‘rapid biomarker-based non-sputum-based test for detecting tuberculosis’ and a triage test, the significant improvement after careful fine-tuning is encouraging for the future of CAD for paediatric tuberculosis. The divergence of CAD4TB scores in ‘tuberculosis’ and ‘not tuberculosis’ cases after fine-tuning (Fig 4) as well as the improved ability to identify lymphadenopathy on the heat maps (Fig 5) indicate specific areas of improvement using a relatively small training set. Replication of this fine-tuning approach on a larger scale is likely to further improve performance without the need for the prohibitively large number of chest x-rays that would be required if a paediatric model was built *de novo*. A larger chest x-ray dataset would also allow for a more nuanced exploration to identify which subsets of radiological abnormalities predict higher CAD scores which could prove useful in the software development process. A challenge to ongoing CAD development remains the limited access to appropriate paediatric datasets. The curation of accurately labelled chest x-ray datasets from children with well-characterised tuberculosis disease phenotypes that are globally representative and accessible to CAD developers is a priority and will require collective effort. There also needs to be careful technical consideration around training CAD software to evaluate the large airways and the mediastinal structures on chest x-rays from children, as well as efforts to automate interpretation of lateral chest x-rays.

In this first publication on the use of CAD to identify ‘tuberculosis’ on chest x-ray from children, we demonstrate that CAD performance can be meaningfully improved by fine-tuning an existing algorithm with as little as several hundred well-classified paediatric chest x-rays. This work should be replicated on a larger scale and within a broader patient population and studies should be done to investigate how CAD scores can be incorporated into current paediatric tuberculosis treatment-decision algorithms. CAD could offer an alternative to a human read chest x-ray and potentially provide an additional tool for paediatric tuberculosis diagnosis, and thus contribute towards decreasing tuberculosis-associated morbidity and mortality in children.

## Supporting information

S1 Checklist (DOCX)
